# Entire State Control

**Published:** 1921-01-29

**Authors:** 


*To the Editor of* THE HOSPITAL.

### Entire State Control.

*To the Editor of* THE HOSPITAL.

SIR,—As one of the great body of laymen to whom you appeal in your leading columns for an expression of opinion as to the kind of medical service the public needs to-day, allow me very briefly to state the case.

I come into contact with large numbers of people who, in the material circumstances of life, are rather more than just above the class of patient who come within the panel service. You may be surprised to learn how many of them share the view that I now put forward. It is this: Scrap the present panel system in its limited and inefficient application; have nothing to do with dividing lines, but devise a bold scheme of State medical service, which shall be open to every English citizen, from the duke to the dustman. To the middle classes, especially to that numerous element in it which lives by a profession, the increasing cost of keeping in health or dealing with sickness when it comes is growing to be one of the most absorbing anxieties of life; and I do not think the problem will ever be solved for them or for the public at large, except by bringing the medical profession entirely under State control. The prospect of nationalising medical service may frighten the men of medicine; but I believe that in the end such a solution will be as beneficial to the medical profession as to the general community. A. J. C.

Finchley, January 24, 1921.

